# Enriched Population of PNS Neurons Derived from Human Embryonic Stem Cells as a Platform for Studying Peripheral Neuropathies

**DOI:** 10.1371/journal.pone.0009290

**Published:** 2010-02-18

**Authors:** Moran Valensi-Kurtz, Sharon Lefler, Malkiel A. Cohen, Michal Aharonowiz, Rachel Cohen-Kupiec, Anton Sheinin, Uri Ashery, Benjamin Reubinoff, Miguel Weil

**Affiliations:** 1 Department of Cell Research and Immunology, Tel Aviv University, Tel Aviv, Israel; 2 Department of Neurobiology, Tel Aviv University, Tel Aviv, Israel; 3 Department of Gynecology, Hadassah University Hospital, Jerusalem, Israel; University of Southern California, United States of America

## Abstract

**Background:**

The absence of a suitable cellular model is a major obstacle for the study of peripheral neuropathies. Human embryonic stem cells hold the potential to be differentiated into peripheral neurons which makes them a suitable candidate for this purpose. However, so far the potential of hESC to differentiate into derivatives of the peripheral nervous system (PNS) was not investigated enough and in particular, the few trials conducted resulted in low yields of PNS neurons. Here we describe a novel hESC differentiation method to produce enriched populations of PNS mature neurons. By plating 8 weeks hESC derived neural progenitors (hESC-NPs) on laminin for two weeks in a defined medium, we demonstrate that over 70% of the resulting neurons express PNS markers and 30% of these cells are sensory neurons.

**Methods/Findings:**

Our method shows that the hNPs express neuronal crest lineage markers in a temporal manner, and by plating 8 weeks hESC-NPs into laminin coated dishes these hNPs were promoted to differentiate and give rise to homogeneous PNS neuronal populations, expressing several PNS lineage-specific markers. Importantly, these cultures produced functional neurons with electrophysiological activities typical of mature neurons. Moreover, supporting this physiological capacity implantation of 8 weeks old hESC-NPs into the neural tube of chick embryos also produced human neurons expressing specific PNS markers *in vivo* in just a few days. Having the enriched PNS differentiation system in hand, we show for the first time in human PNS neurons the expression of IKAP/hELP1 protein, where a splicing mutation on the gene encoding this protein causes the peripheral neuropathy Familial Dysautonomia.

**Conclusions/Significance:**

We conclude that this differentiation system to produce high numbers of human PNS neurons will be useful for studying PNS related neuropathies and for developing future drug screening applications for these diseases.

## Introduction

Stem cells are unique cells that have the capacity for self renewal, exhibit pluripotency and can proliferate indefinitely in culture while maintaining epigenetic and karyotypic stability [1] . Differentiation of human embryonic stem cells (hESC) into neurons can occur spontaneously, however it is not efficient and the resulting neurons make only a small fraction of the total cell population [2]. By contrast, derivation of Neural Progenitors (NPs) from hESC, in the presence of Noggin as a pre-step for differentiation, yielded high numbers of neurons of various subtypes [3]. These human NPs (hNPs) were capable of extensive proliferation *in vitro* and expressed early neuroectoderm markers [3]. Prolonged propagation of hNPs shifted their differentiation potential from neuronal to glial fate, enabling them to differentiate into astrocytes and oligodendrocytes, [4]. A number of studies have successfully obtained different neuronal subtypes by applying various differentiation protocols [5], [6], while others have shown that hESC can efficiently produce motoneurons (MN) that could serve as tools for the study of MN degenerative diseases [7], [8], [9]. So far, the potential of hESC-NPs to differentiate into PNS derivatives resulted in a relatively low yield of PNS neurons [10], [11], [12]. Here, we describe a better procedure to derive mature and functional peripheral neurons, *in vitro*, from hESC-NPs. These NPs-derived PNS neurons can serve as a platform for studying PNS-related neuropathies such as Familial Dysautonomia (FD) which affects the normal development and survival of the sensory and autonomic nervous system [13], [14]. And so, using this differentiation system, we show for the first time in human PNS neurons the normal pattern of expression of the protein IKAP/hELP1, in which a splicing mutation of the gene encoding this protein causes FD [15], [16], [17]

## Results

### Characterization of Crest-Derived Markers in hNPs Prior to Final Differentiation

**Figure 1:**
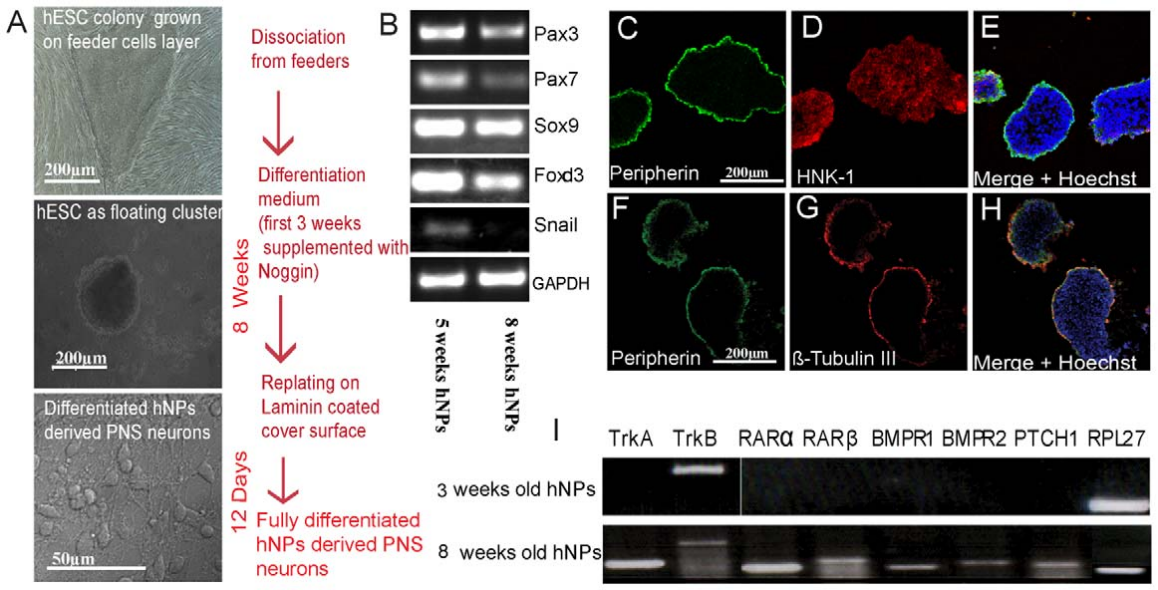
hESC derived NPs express neural crest PNS markers and PNS related growth factor receptors. (A) Schematic flow diagram of the differentiation protocol of hESC derived NPs along 10 weeks culture. (B) RT-PCR analysis of mRNA extracts from hNPs at 5 and 8 weeks in suspension cultures express various early neural crest specific markers prior to final differentiation. Expression of the housekeeping gene GAPDH served as reaction control for each sample. (C–H) Cross section of 5 weeks old NPs in suspension cultures expressing the migratory-crest marker HNK-1 throughout the sphere section (D), while at the edge of the sphere section colocalization of the PNS neuronal marker peripherin and the pan neuronal marker β-Tubulin III is observed (C, F and G respectively). Scale bars are indicated in A and C. (I) Comparative RT-PCR analysis of mRNA extracts from hNPs at 3 and 8 weeks in suspension cultures express different pattern of neuronal differentiation-related growth factor receptors. Expression of the housekeeping gene RPL-27 served as reaction control for each sample.

Spheres enriched for NPs were derived according to our previously published protocol [18] by culturing hESC clusters for 3 weeks in suspension in a chemically defined medium supplemented by FGF2 and noggin. The clusters were further propagated as spheres in the same medium supplemented by FGF2. We have previously shown (Itsykson et al., 2005) that these spheres were mainly comprised of NPs, which express neural markers such as PSA-NCAM, NCAM, Musashi and A2B5. We sought to characterize the potential of these spheres to give rise to PNS neurons. To this end, at the end of 5 weeks of sphere propagation (8 weeks from derivation) the hNPs were placed on laminin coated glass coverslips for a period of 12 days to allow terminal neuronal differentiation (Figure 1A). In order to reveal whether hESC derived NPs have the potential to produce PNS derivatives we analyzed the expression of several early and late PNS markers in hNPs at the prime of their neuronal potential as explained above. First, we investigated the expression of several known neural crest-related markers such as: FoxD3, Sox-9, Pax-3, Pax-7 and Snail transcription factors [19], [20]. This was done by RT-PCR analysis of RNA extracts from hNPs of 5 and 8 weeks suspension cultures respectively (Figure 1B). These results confirm that the hNPs spheres in suspension are neural crest specified at this time in culture. Moreover, immunofluorescence staining of 5 weeks hNPs cryosections (Figure 1C–G). showed that the expression of the neural crest marker HNK-1 is localized within the neurosphere (Figure 1D–E) while more mature PNS markers such as Peripherin and βIII-tubulin (Tuj1) are expressed only at the periphery of the hNPs section (Figure 1C,F and E, G respectively). These results may suggest that most cells in the hNPs remain in the PNS precursor state while some intrinsic PNS differentiation is taking place at the periphery of the neurosphere. To further elucidate this possibility we investigated under these culture conditions the expression of some of the relevant growth factor receptors known to be required for signal transduction pathways relevant in specification and commitment to PNS neuronal differentiation [21]. Figure 1I shows representative RT-PCR comparative analyses of 3 and 8 weeks old NPs for the expression of the following growth factor receptors such as receptors for retinoic acid (RARa and RARb), bone morphogenetic protein receptors (BMPR1 and BMPR2), sonic hedgehog receptor (PTCH1) and neurotrophins (TrkA and TrkB). The housekeeping gene RPL 27 was used as positive reaction control. Interestingly, at 3 weeks in culture hNPs do not express most of these receptors while at 8 weeks hNPs, all of these signaling receptors are expressed at this time point. These results indicate that at the time point of 8 weeks from derivation hNPs are primed for terminal PNS differentiation.

### *In Vitro* Differentiation of Human NPs into Peripheral Neurons

**Figure 2:**
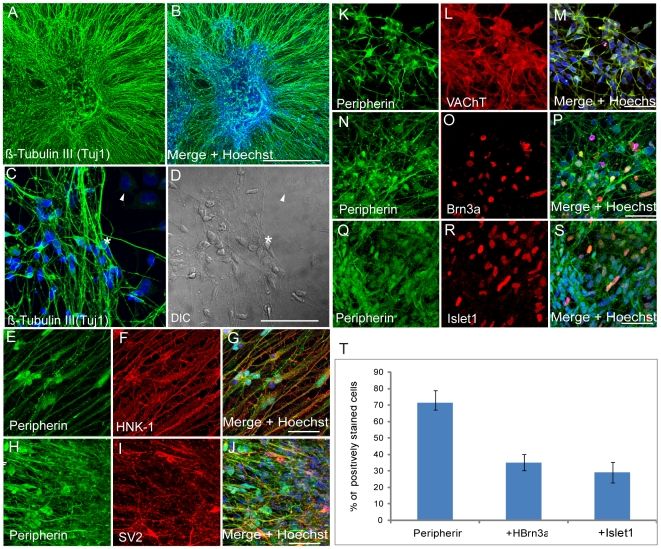
*In vitro* PNS differentiation capacity of hESC derived NPs. (A–S) Eight weeks old Human NPs cultured on laminin for 12 days showing extensive neurite outgrowth expressing the pan neuronal marker β-tubulin III (A–D). These cells also express the PNS marker Peripherin (H,H,K,N and Q) together with the neural crest marker HNK-1 (F), the synaptic vesicle marker SV2 (I) and vesicular acetylcholine transporter VAChT (L), indicative of their maturity, the PNS markers Brn3a (O) and Istet-1 (R). (T) Quantitative analysis from these experiments (N–R) of the proportion of peripherin positive cells from total cells in the culture and the relative numbers of double stained Peripherin+/Brn3a+ and Peripherin+/Islet-1+ from total Peripherin+ cells. (Scale bar: in A-B = 0.5 mm; in C-S = 50 µm).

In light of our observation that hNPs cultures prior to final differentiation express bona fide premigratory and migratory crest markers and relevant signaling components for PNS development we decided to test the potential of these cells to produce PNS neurons by creating suitable conditions for neuronal differentiation in culture. It has been shown that exogenous laminin promotes neuronal differentiation conditions such as survival, migration, neurite outgrowth and synapse formation [22]. We therefore plated 8 weeks old hNPs in suspension cultures onto a matrix surface, coated with poly-D-lysine and laminin, in the presence of the same culture media as described above containing 20 ng/ml bFGF. The results from these experiments are shown in Figure 2. Following two weeks in culture the hNPs developed into neurons expressing the post mitotic pan neuronal marker, Tuj1 (Figure 2A–D). These cells were observed in clusters with their extensions processes migrating away from the center (Figure 2A and B) or as individual neurons with typical round soma and bipolar extensions (Figure 2C and D). The PNS neuronal marker peripherin was also expressed in these cells (Figure 2E, H and K) together with the neural crest lineage marker HNK-1 (Figure 2F). The vast majority of neurons that express peripherin also express the synaptic vesicle marker SV2 (Figure 2I) and express the vesicular acetylcholine transporter (VAChT) (Figure 2L). VAChT is a key element in acetylcholine neurotransmission which is commonly expressed throughout the sensory lineage of the PNS [23]. These results strongly indicate that hNPs are capable to produce PNS neurons with the appropriate synaptic machinery as an indication of neuronal functional maturity. In order to further characterize these differentiated PNS neurons we performed double labeling immunofluorescence analysis using combinations of peripherin with Brn3a antibodies (Figure 2N–P) or peripherin with Islet-1 antibodies (Figure 2Q–S) in order to establish their sensory identity as described previously [24]. Quantitative analysis of these experiments (Figure 2T) revealed that most of the cells in these cultures express peripherin (70% from total cells). About 30% of peripherin positive cells express either the transcription factor Brn3a or Islet-1 confirming independently their sensory neuronal fate. We did not observe in these experiments any expression of the motor neuron specific transcription factor Hb9, strongly indicating that the islet-1 expression is dorsal specific which is related with PNS sensory differentiation in the cultures and not with the ventral motor neuron fate. Additional results from these experiments show that these PNS neurons (peripherin positives) also express the transcription factors Pax3 and Pax 7 further indicating the dorsal restricted PNS differentiation potential of the cells (Figure S1A–F).

Further analysis of the peripherin positive population in these cultures revealed that only few (3–5%) peripherin positive neurons coexpress tyrosine hydroxylase (TH) (Figure S2A–C) indicating a sympathetic neuronal fate as described previously [24]. Overall these results demonstrate that at the 8 weeks time point hESC derived NPs cultured for two weeks on laminin matrix produce highly enriched dorsal PNS neuronal cultures.

### Electrophysiological Recordings of NPs Derived PNS Neurons

**Figure 3:**
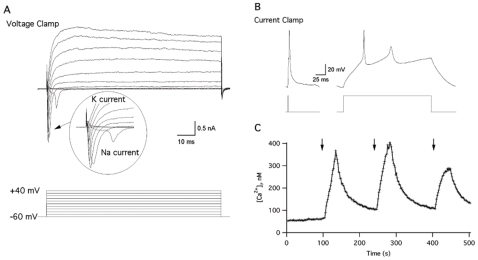
Electrophysiological analysis of NPs derived PNS neurons. The current-voltage (IV) relationship acquired from cell in voltage clamp mode, holding potential was −60 mV (A, upper panel). The inward current deflections are the sodium currents evoked by 200 ms-long step depolarization from −60 to +40 mV with a 20 mV increment (A, lower panel). Representative traces of single action potential (B, left panel) and repetitive action potential firing (B, right panel) acquired from the cell in current clamp mode by 2 ms and 200 ms-long suprathreshold current injection (B, left and right lower panels, respectively). Increase in intracellular calcium concentration evoked by high KCl application (C), Representative trace from fura-2 AM-loaded cell in response to local 70 mM KCl application is indicated by arrows. These experiments were repeated 4 times, using cells from different cultures each time, obtaining the same results.

Besides expressing neuronal markers including SV2 and VAChT, as shown above, these cells show clear mature neuronal morphology that may also indicate neuron functionality. To examine the functional maturity of our hNPs derived neurons we performed electrophysiological recordings using the whole-cell patch clamp technique and intracellular calcium measurements using the FURA-2AM assay. For these analyses 8 weeks old NPs were seeded onto poly-D-Lysine and laminin coated cover slips followed by incubation for 14 days, until they reached 10 weeks in culture, as described above. Human NPs-derived neurons were chosen for recording based on their morphology, exhibiting well developed neuronal shapes. We then performed a series of electrophysiological recording from these cells. Cells were held in the “voltage-clamp” mode and 100 ms depolarization pulses to increasing voltages were applied. As can be seen from Figure 3A, when the cells were depolarized to above −20 mV, they responded with both fast sodium and delayed potassium currents. Moreover, when the cells were hold in the current clamp mode, a short depolarization pulse elicits an action potential (Figure 3B). These results demonstrate that the NPs derived cells differentiated into electrophysiologically mature neurons. We next examined if these cells exhibit calcium influx following depolarization, a process that is crucial for neurotransmitter release. Intracellular calcium measurements were performed by loading the cells with the fluorescent indicator, Fura-2AM, concomitantly with the application of high a KCl solution. This experiment revealed transient changes in calcium influx within the tested cells (Figure 3C). The KCl application caused membrane depolarization, and as a consequence, opening of voltage-dependent calcium channels. As a result, the intracellular calcium concentration increased; hence, indicating functionality of voltage-dependent calcium channels which is essential for neurotransmitter release. Altogether, these results indicate that the NPs-derived PNS neurons developed essential physiological machineries of functional mature neurons.

### Implantation of hNPs into the Developing Neural Tube of Chick Embryos

**Figure 4:**
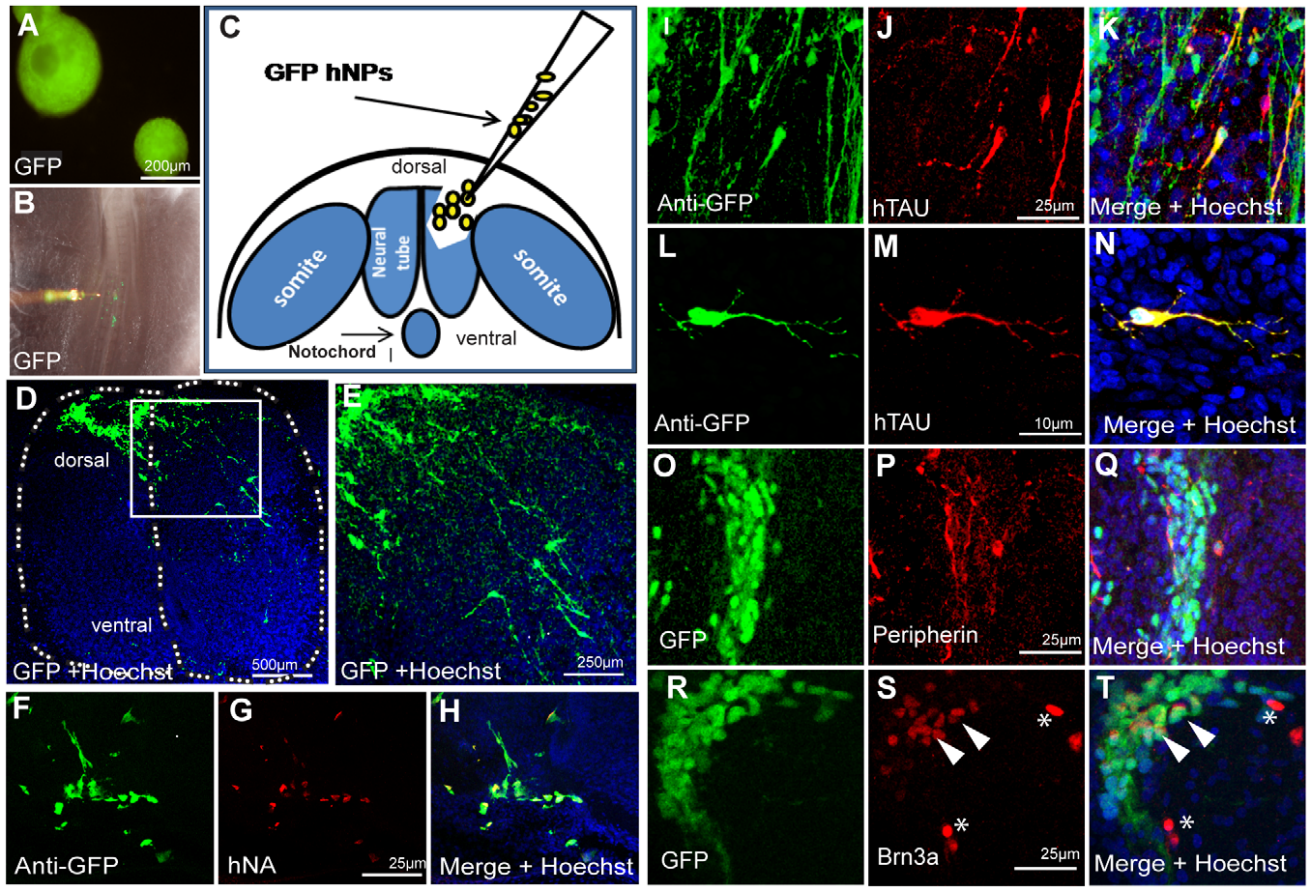
*In vivo* PNS differentiation of 8 weeks old hNPs implanted into the chick developing neural tube. (A) Eight weeks old hESC expressing GFP derived NPs in suspension before cell dissociation. (B) Illustration of the cell transplantation procedure in the dorsal neural tube after removal of one neural fold. (C) Micrograph showing microinjection of GFP+ cells into the implantation site. (D–T) hNPs GFP+ derived cells 7 days after implantation located at the dorsal spinal cord showing extensive migration and neurite outgrowth (D and in high magnification in E). (F–H) implanted human NPs derived cells are specifically identified with anti-GFP antibodies (F) and with anti human specific nuclear antigen (G). Implanted hNPs express the human specific neuronal microtubule-associated protein Tau (I–K, and in high magnification in L–N). These cells also express the PNS markers, Peripherin (O–Q) and Brn3a (Q–S), Scale bars are indicated in representative images.

To strengthen the physiological relevance of the hNPs-derived PNS neurons produced in culture we tested the potential of hNPs to produce human PNS neurons within the developing nervous system of the chick embryo. Implantation experiments were performed *in ovo*. Eight weeks old GFP-hNPs cells derived from the GFP-HES1 cell line [25] were microinjected at the neural tube (NT) trunk level of 18–23 somite chick embryos after removing a portion of one neural fold as described in Figure 4A–C. Implanted GFP-hNPs cells were visualized 7 days following implantation in spinal cord transverse sections. Figure 4D–T shows representative staining of transplantation experiments. The implanted human GFP expressing cells were visualized directly in the dorsal spinal cord (Figure 4D and magnified area in E) or indirectly using anti GFP antibodies (Figure 4F, I and L). Antibodies against human-specific nuclear antigen were used to stain selectively the human nuclei in order to confirm the human identity of the GFP-implanted cells by immunofluorescence analysis (Figure 4F–H). The neuronal identity of these cells was confirmed by applying human-specific neuronal microtubulin associated protein (hTAU) antibodies (Figure 4J–K) The typical neuronal morphology of these cells is exemplified in Figure 4L–N showing co-expression of GFP and hTAU proteins in one hNPs derived human neuron within the chick dorsal spinal cord. To characterize the PNS identity of the implanted GFP-derived human cells we used anti peripherin antibodies, which do not react with the host tissue, (Figure 4O–Q) and the sensory specific marker, the transcription factor Brn3a, that crossreacts with the chick orthologe (Figure 4R–T). Importantly, the human derived PNS cells were located spatially and temporally where PNS neurons are being formed in the chick host at the dorsolateral aspect of the spinal cord as judged by endogenous chick Brn3a expression detected in proximity to the human cells (red labeled nuclei in Figure 4S and T, indicated by arrowheads) or together with human cells expressing GFP (red nuclei within GFP cells Figure 4 RT, indicated by asterisks). Altogether, these results confirm our suggestion that the implanted hNPs have the developmental potential to produce PNS neurons *in vivo* after seven days of implantation in time and place when the host's PNS neurons are formed.

### IKAP Expression in Human PNS Neurons

Having in hand an experimental system to produce human PNS neurons in high numbers prompted us to use this unique platform to characterize IKAP/hELP1 expression within PNS neurons as future model to study FD. To this end we performed immunofluorescence analysis of NPs derived PNS neurons, in vitro and in vivo, as described above, using commercially available IKAP antibodies.

**Figure 5:**
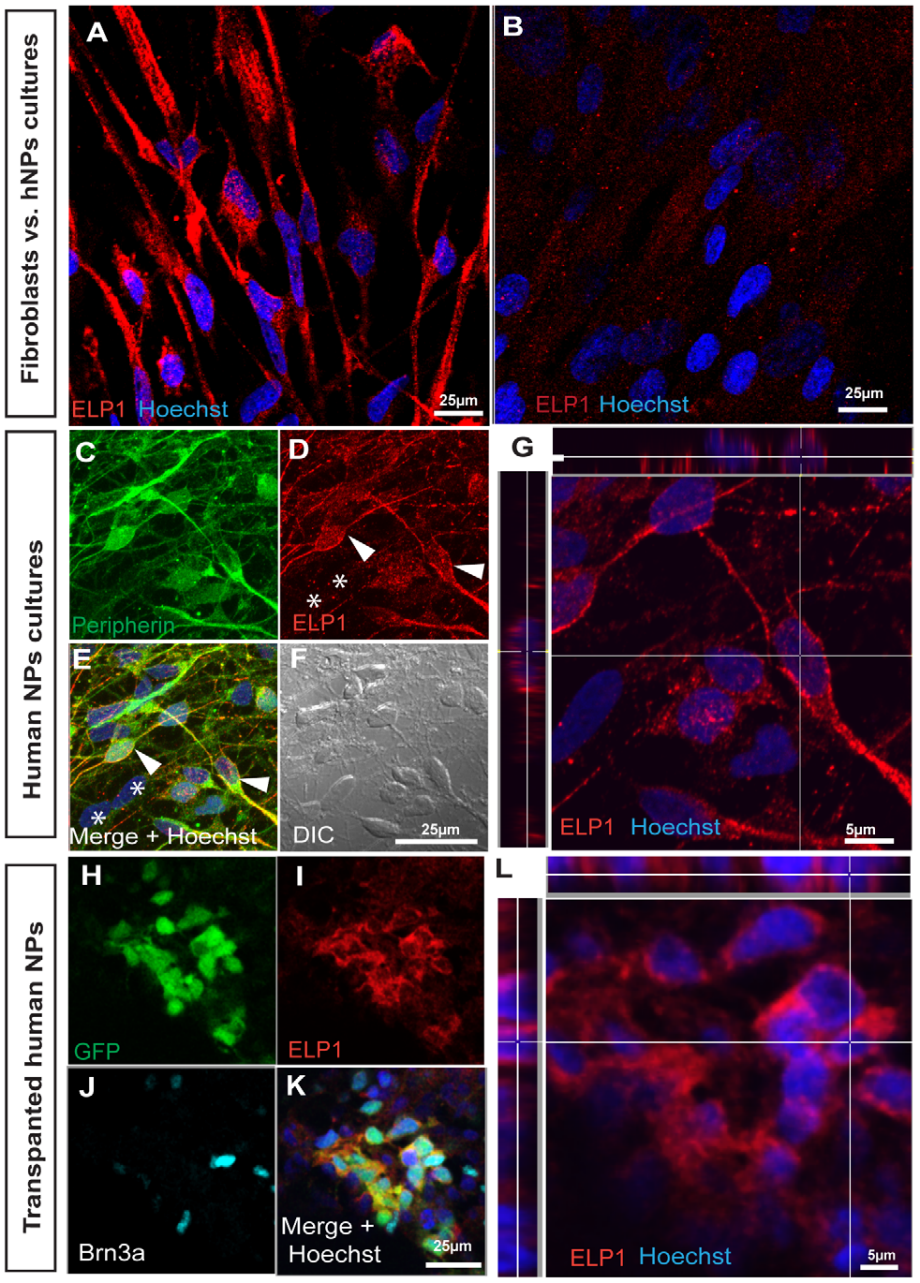
Characterization of IKAP/hELP1 expression in hNPs derived PNS neurons *in vitro* and *in vivo*. (A–B) comparative analysis of the expression of IKAP/hELP1 in hNPs derived PNS cultures (A) and in human fibroblasts (B) under the same confocal microscopy settings, showing higher levels of IKAP/hELP1 in the hNPs derived neurons. (C–F) Confocal micrographs showing the expression of IKAP/hELP1 in hNPs derived PNS neurons in vitro as judged by Peripherin positive staining. Peripherin+ cells show high levels of IKAP/ELP1 (D and, indicated by arrowheads) in contrast to non-peripherin expressing cells in the culture (fig. 5D and E, indicated by asterisks). (G) Magnified area of the same confocal plane (C–F) showing orthogonal analysis of IKAP/hELP1 localization mainly in the cytosol of PNS neurons. Confocal micrographs showing the expression of IKAP/hELP1 in GFP+ hNPs derived PNS neurons in vivo, which express Brn3a, at the implanted chick spinal cord (H–K). (L) Magnified area of the same confocal plane (H–K) showing orthogonal analysis of IKAP/hELP1 localization mainly in the cytosol of human PNS neurons in vivo. Scale bars are indicated in representative images.

We detected IKAP/hELP1 expression in PNS neurons as shown in Figure 5. IKAP/hELP1 was expressed in the cytoplasm of peripherin expressing neurons. In comparison to non-neuronal cells, like human fibroblasts, IKAP/hELP1 expression in PNS neurons seems to be much higher as judged by the intensity of the monoclonal antibody staining under the same confocal scanning conditions (Figure 5A and B). It should be noted that, IKAP/hELP1 expression in human PNS neurons (peripherin positives) seems to be more granular and localized along the axons and dendrite terminals (Figure 5C–E, indicated by arrowheads) in contrast to non-peripherin expressing cells in the culture (Figure 5C–E, indicated by asterisks). According to previous studies IKAP/hELP1 was found to be part of the Elongator complex involved in RNA transcription process in yeast and mammalian cell lines [26], [27] therefore we performed in more detail a confocal localization analysis of IKAP/hELP1 in PNS neurons. We did not observe any significant expression of IKAP/hELP1 in the nuclei of these PNS neurons (shown as orthogonal section in Figure 5G which is a field magnification from 5D). Similar results of IKAP/hELP1 expression and intracellular localization were obtained with implanted GFP-hNPs derived PNS neurons *in vivo* as judged by coexpression of IKAP/ELP1 with Brn3a positive nuclear staining, respectively (Figure 5H–K and magnified orthogonal section 5L). Moreover, we have also observed similar cytoplasmic localization of IKAP/hELP1 in hTAU positive hNPs derived neurons *in vivo* (Figure S3A–F). Altogether, these observations may indicate that IKAP/hELP1 is differently regulated in PNS neurons and other cell types. This may support the assumption that IKAP/hELP1 plays a special role in PNS neurons. This assumption is in accord with the fact that the FD mutation of the IKAP gene selectively affects PNS development and postnatal PNS neurons.

## Discussion

Here we describe a unique *in vitro* experimental system that allows the study of PNS related diseases such as FD. So far, most studies related to NPs derived from hESC in culture, have focused on the Central Nervous System (CNS) and motoneurons derivatives yielding moderate enrichment of dorsal PNS neurons [8], [28], [29], [30]. Here we demonstrate that hESC-derived NPs produce highly enriched PNS neuronal cultures under defined medium conditions. Our experiments show that 5 weeks old NPs, in suspension culture, express already neural crest specific markers [19], [20] as well as signaling growth factor receptors [21] that are essential for PNS development. However at this early time point, following two weeks of differentiation on laminin coated surface, we have observed the formation of neuronal rosettes and the intracellular miss localization of Islet1 transcription factor in the cytosol of these cells (see Figure S4). This rosette formation is typical of early stages of hESC neural differentiation and was used previously as a source to generate motoneurons [7], [31]. This evidence could explain the fact that we have not detected any Hb9 expression in our differentiation protocol at more mature stages using 8 weeks old hNPs to generate PNS neurons. Together with this our results show Islet 1 expression concomitantly with Pax3 and Pax7 expression in differentiated PNS neurons indicating strong dorsalization of the hNPs cultures. Interestingly, while the expression Pax3 and Pax7 is maintained in places of the developing mouse PNS [32], in the developing chick embryo it becomes down regulated during terminal neuronal differentiation [33]. In our differentiated hNPs cultures we have detected high levels of these transcription factors concomitant with the expression of terminal differentiated markers. It appears that Pax3 and Pax7 expression at 10 weeks of hNPs differentiation *in vitro* resembles more to the *in vivo* mouse model rather than that of the chick. We believe that this observation together with the expression of crest specific markers can explains the commitment of our differentiated hNPs to yield a high proportion of peripherin expressing neurons following a total of 10 weeks in culture. Moreover, thirty percent of these cells co-express typical sensory protein markers such as Brn3a and Islet-1 suggesting that under these culture conditions at least sensory neuronal commitment takes place. Further evidence supports this view that most of the peripherin positive cells in our cultures express VAChT which is known to be present in cholinergic neurons of the PNS [34]. We can therefore conclude that the PNS enrichment in culture is due to an intrinsic developmental capacity of the hESC derived-NPs to produce PNS precursors. Proper culturing conditions apparently promote differentiation of these cells to PNS neurons *in vitro*. The importance of the *in vitro* differentiation system described here is supported by our demonstration that the PNS cultures produce mature and functionally active neurons as judged by the expression of synaptic vesicle proteins and by electrophysiological analyses. In addition we have also demonstrated the physiological PNS potential of 8 weeks old NPs 7 days after implantation of the cells into the developing neural tube (NT) of 18–23 somite chick embryos *in ovo*. The cells were implanted into the developing neural tube at the trunk region where neural crest will eventually give rise to almost the entire PNS [35], [36]. The human implanted cells showed in the spinal cord, 7 days after implantation, neuronal-like morphologies expressing neuronal and PNS specific markers. The transplanted hNPs were implanted at a time and location were neural crest cells delaminate and migrate toward the periphery. However in our experiments most of the transplanted cells remain at the dorsal part of the spinal cord and did not migrate to the periphery. We have obtained peripheral neurons in the dorsal aspect of the neural tube, this is most probably due to the intrinsic differentiation potential of the 8 weeks old hNPs combined with the host environment at the time of implantation. This can be implied by our observation that the implanted human neurons express Brn3a at the site where the host expresses Brn3a. This *in vivo* differentiation of human NPs driven by host signals should depend as well on the expression of the relevant growth factor receptors by the human cells. Characterization of the expression pattern of different growth factors receptors in hNPs in culture showed that 8 weeks old hNPs, as opposed to 3 weeks old hNPs express several basic signaling receptors required for responding to different growth factors which are responsible of PNS differentiation. The most relevant receptors for PNS development that we have detected in human NPs include the Sonic hedgehog receptor PTCH1, neurotrophin receptors TrkA and TrkB, and the BMP receptors BMPR1 and BMPR2, which are known to be involved, in proliferation and survival of sensory and autonomic neurons [37], [38], [39]. Having in hand the way to produce rich populations of PNS neurons derived from hESC, allows us to study, IKAP/hELP1 expression in the type of neurons most affected in Familial Dysautonomia. We observed that IKAP/hELP1 is expressed in neurons differently than its expression in fibroblasts indicating a difference in the levels and pattern of its expression. Although, IKAP/hELP1 is considered to be a component of the transcription elongator complex we failed to detect IKAP/hELP1 staining in the nucleus of fibroblasts or of the PNS neurons with the IKAP/hELP1 monoclonal antibody used. This is in accord with previous reports showing cytoplasmic localization of IKAP in mouse embryonic fibroblasts and Hela cells using three different IKAP antibodies as well as by IKAP overexpression [40]. Altogether, our results may support the assumption that IKAP/hELP1 plays a special role in PNS neurons. This assumption is consistent with the fact that the FD mutation of the IKBKAP gene selectively affects PNS development and postnatal PNS neurons. These results are a step forward towards understanding IKAP/hELP1 role and its implication to the FD phenotype. The *in vitro* and *in vivo* experimental PNS differentiation system that we have described is aimed to study IKAP/hELP1 in PNS neurons and to explain the FD phenotype. In addition, we believe that this system could also be a useful tool for future drug screening using PNS neurons derived from FD induced pluripotent stem cells as well as for the study of other peripheral neuropathies.

## Materials and Methods

### Cell Lines and Cultures

Human Neural Precursors (hNPs) were derived from ‘HES1’ hESC line [2] or from genetically modified HES1 cells that constitutively express GFP (‘GFP-HES1’; [25] as described previously [18]. Briefly, HES1 colonies with normal karyotype (46XX) that were cultured on human foreskin feeders were dislodged 7 days after passage using Collagenase IV (Invitrogen Corp., Carlsbad, CA) into small clusters of 1500–2000 cells and transferred into suspension culture dishes at densities of up to 80×103 cell/ml. The HES1 cell clumps were cultured in medium consisting of DMEM/F12 (1∶1), B27 supplement (1∶50), glutamine 2 mM, 50 U/ml penicillin, 50 mg/ml streptomycin (Invitrogen, Carlsbad, CA), 20 ng/ml rm-bFGF(Sigma, St. Louis, MO), and 500–700 ng/ml rm-noggin (R&D Systems Inc., Minneapolis, MN) for 3 weeks, followed by removal of noggin. The formed NP spheres were propagated in the same medium that was refreshed every three to four days. The spheres size was maintained at ≤0.5 mm in diameter by pipetting them through a tip. After noggin removal the NPs were kept in suspension for several weeks in the presence of bFGF2 until attachment onto matrix for terminal differentiation. Foreskin primary fibroblasts (FSK) FSK were cultured in DMEM (Gibco, Invitrogen) supplemented with 10% FCS (Hyclone), 2 mM pyruvate (Gibco, Invitrogen), 50 U/ml penicillin and 50 mg/ml streptomycin, (Beit haEmek biological industries, Israel).

### Neuronal Differentiation

8 weeks old spheres were disrupted into ∼0.1 mm diameter clusters by fine pipettation. 12 mm cover slips (De-Groot Laboratory Equipment LTD, Israel) were incubated with 10 µg/ml Poly-D-Lysine (30–70 kDa, Sigma-Aldrich Corp. Rehovot, Israel) for 1 hour at room temperature. At the end of incubation the cover slips were rinsed once with PBS and incubated with 4 µg/ml Laminin (Sigma-Aldrich Corp. Rehovot, Israel) for overnight at 4°C. The cover slips were rinsed twice with PBS before cells were plated. The small cell clusters were placed on the coated cover slips in the same growth medium (as described above) in the presence of bFGF but without being changed for the differentiation period of 10–13 days.

### Implantation of Human NPs into Chick Embryos

Fertilized White Leghorn chicken eggs were incubated for 48 hours at 38°C until they reached the 18–23 somite stage. 3 µl of Fast Green (Sigma-Aldrich Corp. Rehovot, Israel) was added on top of the embryo and staged in ovo according to Hamburger and Hamilton (HH) [41]. A small incision was made in the vitelline membrane at the level of the last 4 developing somites. At this site, one neural fold (right side) was removed over a length of 4–5 somites by gently tearing the tissue with a pulled sharp glass micropipette. Human NPs were dissociated in 0.05% EDTA/PBS solution and then microinjected into the operated site of the neural tube. The eggs were sealed and incubated for 5 to 7 days. For cryosectioning, embryos were removed from their eggs fixed in 4% paraformaldehyde/PBS for 1–2 hours, then transferred overnight into 30% sucrose/PBS at 4°C and finally embryos were embedded in OCT (Tissue-Tek, Andwin Scientific) for block preparation. Transverse 15 µm cryosections were made along the embryo's implanted area and were stored at −80°C until use for immunoflurescence analysis (as described later for cell culture).

### Reverse Transcription (RT) PCR

Cells in culture were collected using Trypsin-EDTA (Biological Industries, LTD, Beit Haemek, Israel) and from the cell pellets total cellular RNA was isolated using EZ-RNA Total RNA Isolation kit (Biological Industries, LTD, Beit Haemek, Israel) and eluted in 50 µl RNAse-free pure water. The concentration of total RNA was measured using a Nano Drop Spectrophotometer (Nano Drop Technologies, USA). To eliminate a possible contamination with genomic DNA, 0.1 U/µl DNAse I (Ambion) was applied according to the manufacturer's protocol. Total RNA (0.3–0.5 µg) was reverse-transcribed into complementary DNA (cDNA) with Reverse It 1st Strand kit (ABgene) using oligo-dT as a primer according to the manufacturer's instructions. PCR reaction was carried out using ReddyMix Master Mix (ABgene) according to manufacture instruction. PCR products were analyzed by electrophoresis on 2% agarose gel. The primers for the following human genes were used: Primer sequence (5′→3′):

Snail fwd GCTGCAGGAGGACTCT, rev GACAGAGTCCCAGATG; Sox9 fwd AGTGGGTAATGCGCTT, rev CGAAGATGGCCGAGAT;Pax7 fwd GGCGACT CCGGATGTA, rev CGCGGCTAATCGAACT; Pax3 fwd AGCACCCCAATCAGATGAAG, rev TGTCTGGGTTG GAAGGAATC; Foxd3 fwd GTTCATCAGCAACCGC, rev GTCCAGTA GTTGCCCT; GAPDH fwd TTTTACTCTGGTAAAGTGG, rev TTTTGGCTCCCCCCTGCAAAT. BMPR1 fwd GCACCAGAGGATACCTTGC, rev AATGAGCAAAACCAGCCATC; BMPR2 fwd TTCCACCTCCTGACACAACA, rev GGCAATGTTGTCATGTTCCA; RARα fwd CCTCTACCCCGCATCTACAA, rev CGTCAGCGTGTAGCTCTCAG; RARβ fwd GAAACAGGCCTTCTCAGTGC, RARβ rev GGTGACTGACTGACCCCACT; TrkA fwd CACAGAGCTGGAGCAG TCAG, rev AGCGTGTAGTTGCCGTTGTT; TrkB fwd ACCCCCATTCGCATC TAAC, rev CAGAAATGCTTTATGAGCCACA; ShhR (PTCH1) fwd CGCACAG AACTCCACTCAAA, rev GGGCCAGAAGAAAA ACATCA; Human RPL27 fwd ATGATGGCACCTCAGATCG, rev AAGAAC CACTTGTTCTTGCC; Human GAPDH fwd CTTTTACTCTGGTAAAGTGG, rev TTTTGGCTCCC CCCTGCAAAT.

### Immunocytochemistry Analysis

At the end of incubation, the cells grown on cover slips were gently rinsed in PBS and fixed with 4% paraformaldehyde/PBS for 20 minutes. After rinsing in PBS, the cover-slips were incubated for one hour in blocking solution containing 2% bovine serum albumin and 0.05% Triton in PBS. The following antibodies used in this study were diluted in blocking solution containing 2% FCS and 0.05% Triton x-100: Rabbit anti-Peripherin (1∶200), mouse anti-Brn3a (1∶150), chick anti -TH (1∶300), mouse anti-hNA (1∶300), goat anti-VAChT (1∶700) from Chemicon International, Temecula, CA, U.S.A.; anti-SV2 (1∶200), anti-hTAU (1∶200), anti-Islet1 (1∶200) from Developmental Studies Hybridoma Bank, Iowa, U.S.A.; Mouse anti β-III-Tubulin (“TUJ1”, 1∶1000; Covance), rabbit anti-GFP (1∶500, Invitrogen). Mouse anti HNK-1 (1∶100, a kind gift from Dr. Chaya Kalcheim, Hebrew University of Jerusalem). Mouse anti human IKAP/ELP1 (1∶300 BD Biosciences) and rabbit anti human IKAP/ELP1 (1∶300, Anaspec). Secondary antibodies: donkey anti goat IgG-cy5, donkey anti mouse IgG-cy2, donkey anti chick IgG-cy2, goat anti rabbit IgG-cy5 and goat anti mouse cy3 (1∶1000; Jackson ImmunoResearch). Cover slips were incubated with the primary antibodies for 1 hour at room temperature or overnight at 4°C. The secondary antibodies were applied for 1 hour of incubation at room temperature, together with Hoechst 33258 (0.1 µg/ml) to stain cell nuclei. Cover slips were then rinsed in PBS, mounted on microscope slides in gel mount (BD Biosciences), and sealed with nail polish. Confocal analysis of the immnofluorescently labeled preparations was performed with LSM Meta confocal microscope (Carl Zeiss, Oberkochen, Germany). Image analysis was made using LSM software (Zeiss), Slidebook 4.0 (Intelligent Imaging Innovations, Inc), Photoshop (Adobe) and Imaris (Imaris, Bitpalne).

### Electrophysiology and Calcium Measurements

Electrophysiological recordings were performed utilizing EPC-9 patch-clamp amplifier and Pulse v8.31 software (HEKA Electronics Gmbh, Lambrecht, Germany), running on Macintosh PPC 8600/200 computer. The extracellular solution consisted of (mM): NaCl 140, KCl 3, CaCl2 2, MgCl2 1, HEPES 10 (Sigma-Aldrich Corp. Rehovot, Israel), supplemented with 2 mg/ml glucose (Sigma-Aldrich Corp. Rehovot, Israel), pH 7.4, osmolarity adjusted to 340 mOsm. The patch pipettes had resistances of 3–5 MOhm after filling with a solution containing (in mM): KCl 135, HEPES 10, glucose 5, K2ATP 1, MgATP 1 (Sigma-Aldrich Corp. Rehovot, Israel), pH 7.4, osmolarity adjusted to 315 mOsm. Intracellular calcium measurements (FURA assay): The cells were incubated with 3 µM fura-2 acetoxymethyl ester (AM) (Molecular Probes, Inc., Eugene OR, U.S.A) for 1 hour at 37°C and 5% CO_2_ incubator before being used for experiments. To facilitate loading of the AM ester indicators, the mild detergent, pluronic acid F-127 (Molecular Probes, Inc., Eugene OR, U.S.A), was added to fura-AM in a 1∶1 ratio and vortexed thoroughly with 0.5 ml of NPs medium (as described in 2.2) immediately before incubation. After the incubation, cells were washed three times with the external recording solution which contained (mM): NaCl 140, KCl 3, CaCl2 2, MgCl2 1, HEPES 10, supplemented with 2 mg/ml glucose (Sigma-Aldrich Corp. Rehovot, Israel), pH 7.4, osmolarity adjusted to 340 mOsm. The cells loaded with the fura-2 AM indicator were then transferred to an inverted microscope (IX-50; Olympus, Tokyo, Japan) equipped with a x40 objective (UAPO/340; Olympus, Tokyo, Japan). Fura-2 AM was excited at 350 and 380 nm generated by a monochromator (TILL Photonics) and detected through a 500-nm long-pass filter using a photomultiplier tube (TILL Photonics). Exposure times were 20 ms for both 350 and 380 nm; interval between measurements was 2 milliseconds. During the experiments, the cells were perfused continuously with the external solution. After recording the baseline calcium levels for about 100 seconds, the cells were stimulated by 70 mM KCl (Sigma-Aldrich Corp. St. Louis, MO), which was applied locally through a glass pipette. After the application, the pipette was withdrawn from the cell. Intracellular calcium concentration was calculated from the fluorescence ratio R according to the method of Grynkiewicz et al [42].

## Supporting Information

Figure S1 Expression of Pax3 and Pax7 in hNPs derived PNS neurons. (A–F) Eight weeks old human NPs cultured on laminin for 12 days showing extensive neurite outgrowth expressing the PNS marker Peripherin (A, C and D, F) together with the dorsal markers, the transcription factors Pax3 (B and C) and Pax7 (E and F). Scale bars are indicated in representative images.
(1.55 MB TIF)

Figure S2 Expression of Tyrosine hydroxylase (TH) in hNPs derived PNS neurons. (A–C) Eight weeks old human NPs cultured on laminin for 12 days showing extensive neurite outgrowth expressing the PNS marker Peripherin (A and C) together with TH that is expressed by a small number of cells (B and C). Scale bar in C is representative.
(1.83 MB TIF)

Figure S3 IKAP/hELP1 in hNPs derived PNS neurons *in vivo*. (A–E) Confocal micrographs showing the expression of IKAP/hELP1 in GFP+ together with the expression of human specific TAU (hTAU) in hNPs derived PNS neurons in the spinal cord of the chick embryo after 7 days of implantation. (E) Magnified area of the image in (A–D) showing orthogonal analysis of IKAP/hELP1 localization mainly in the cytosol of human PNS neurons *in vivo*.
(2.41 MB TIF)

Figure S4 Five weeks hNPs differentiation culture shows rosettes formation with miss localization of Islet-1. (A–C) Five weeks old hNPs expressing GFP was plated on laminin coated surface for 12 days in culture showing typical rosette structure (A). Note that Islet-1 expression (B) in these cells is cytoplasmic and not nuclear as expected, which may indicate a more neuronal precursor state rather a mature neuron as shown in similar experiments with 8 weeks old hNPs (see Figure 2).
(1.25 MB TIF)
