# Supplementary figures and images for: Enriched Population of PNS Neurons Derived from Human Embryonic Stem Cells as a Platform for Studying Peripheral Neuropathies
Source: PLoS One. 2010 Feb 18;5(2):e9290. doi: 10.1371/journal.pone.0009290 (PMC2823780; doi:10.1371/journal.pone.0009290)

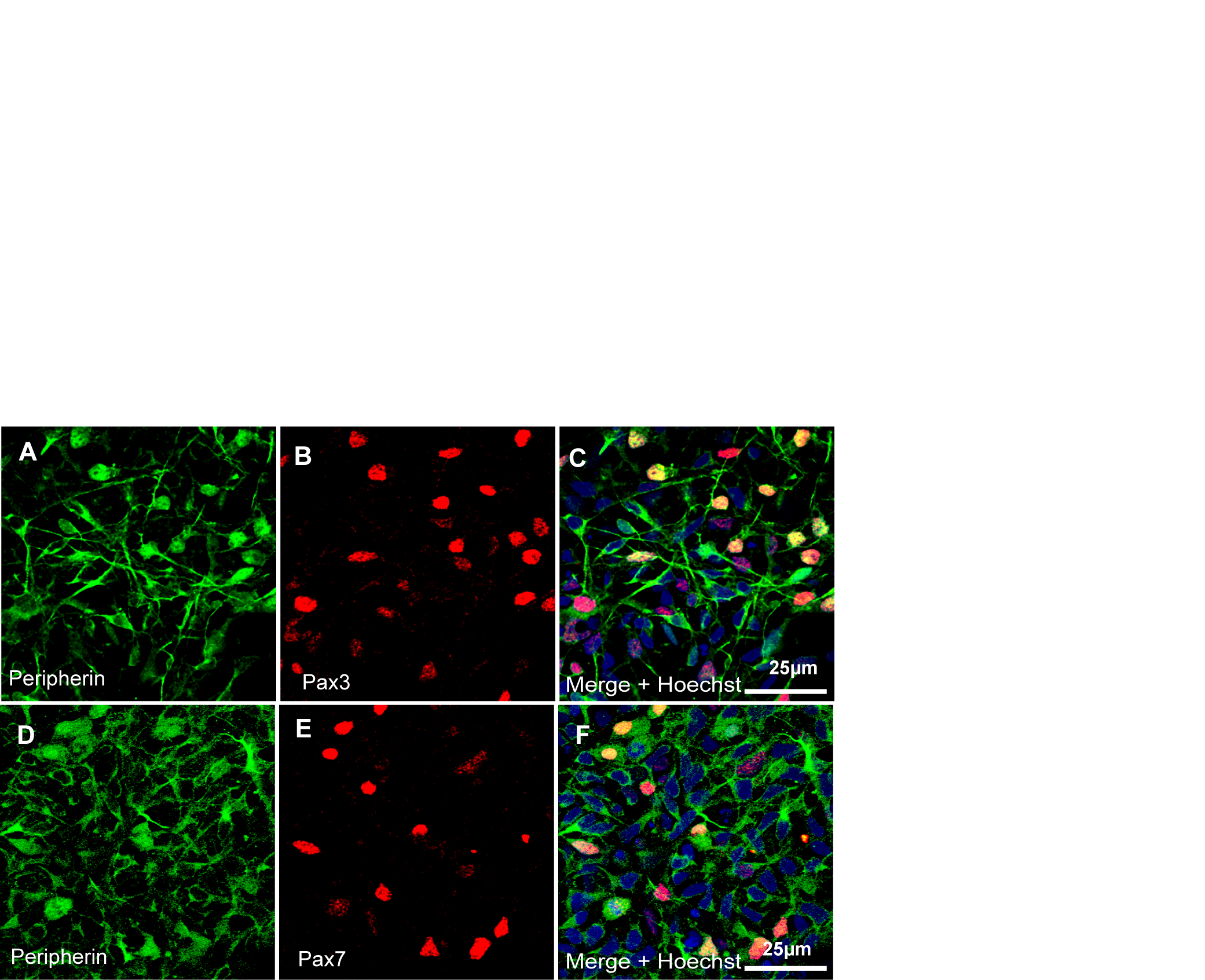

Supplement: Figure S1 — Expression of Pax3 and Pax7 in hNPs derived PNS neurons. (A–F) Eight weeks old human NPs cultured on laminin for 12 days showing extensive neurite outgrowth expressing the PNS marker Peripherin (A, C and D, F) together with the dorsal markers, the transcription factors Pax3 (B and C) and Pax7 (E and F). Scale bars are indicated in representative images. (1.55 MB TIF) [file pone.0009290.s001.tif]

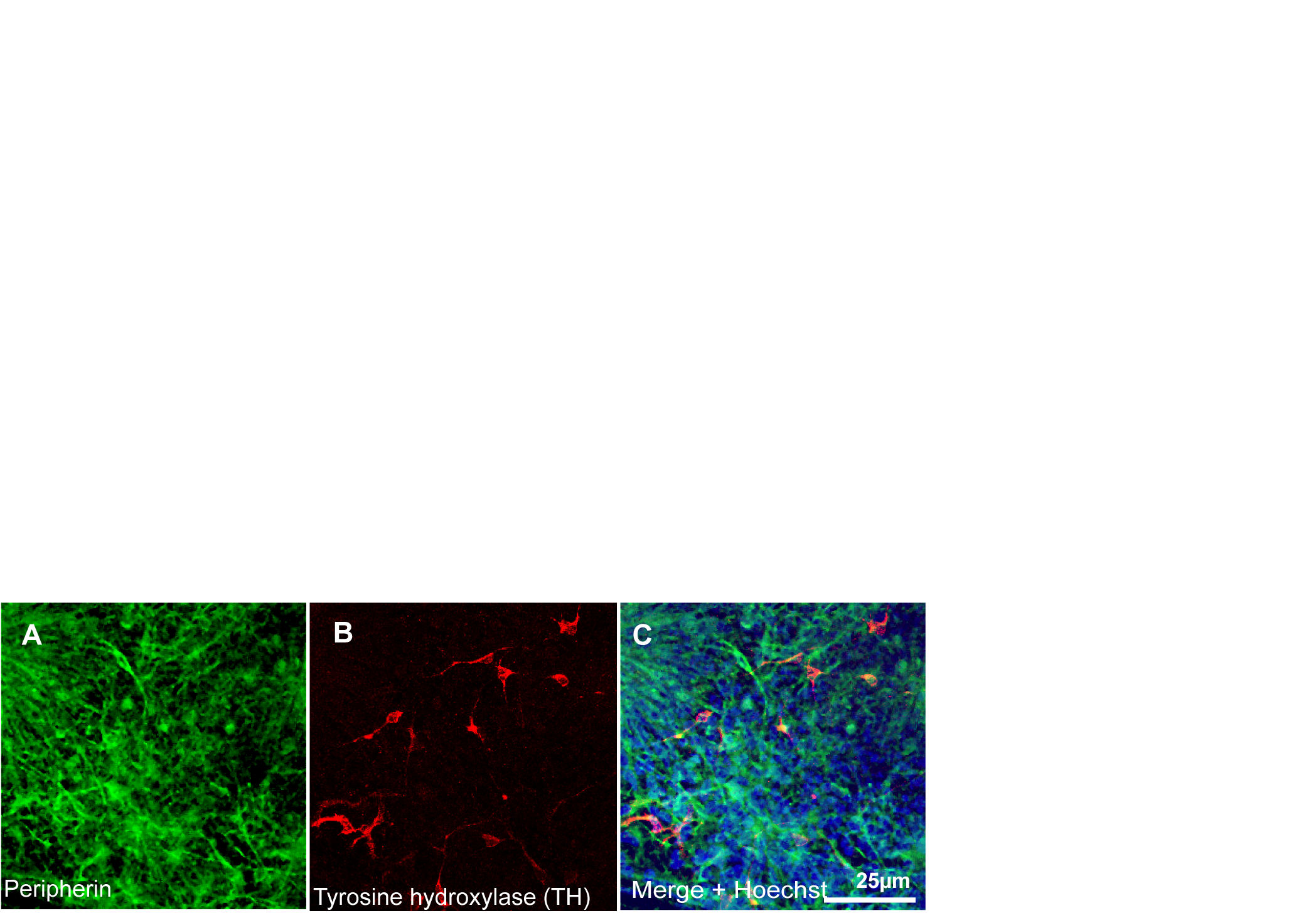

Supplement: Figure S2 — Expression of Tyrosine hydroxylase (TH) in hNPs derived PNS neurons. (A–C) Eight weeks old human NPs cultured on laminin for 12 days showing extensive neurite outgrowth expressing the PNS marker Peripherin (A and C) together with TH that is expressed by a small number of cells (B and C). Scale bar in C is representative. (1.83 MB TIF) [file pone.0009290.s002.tif]

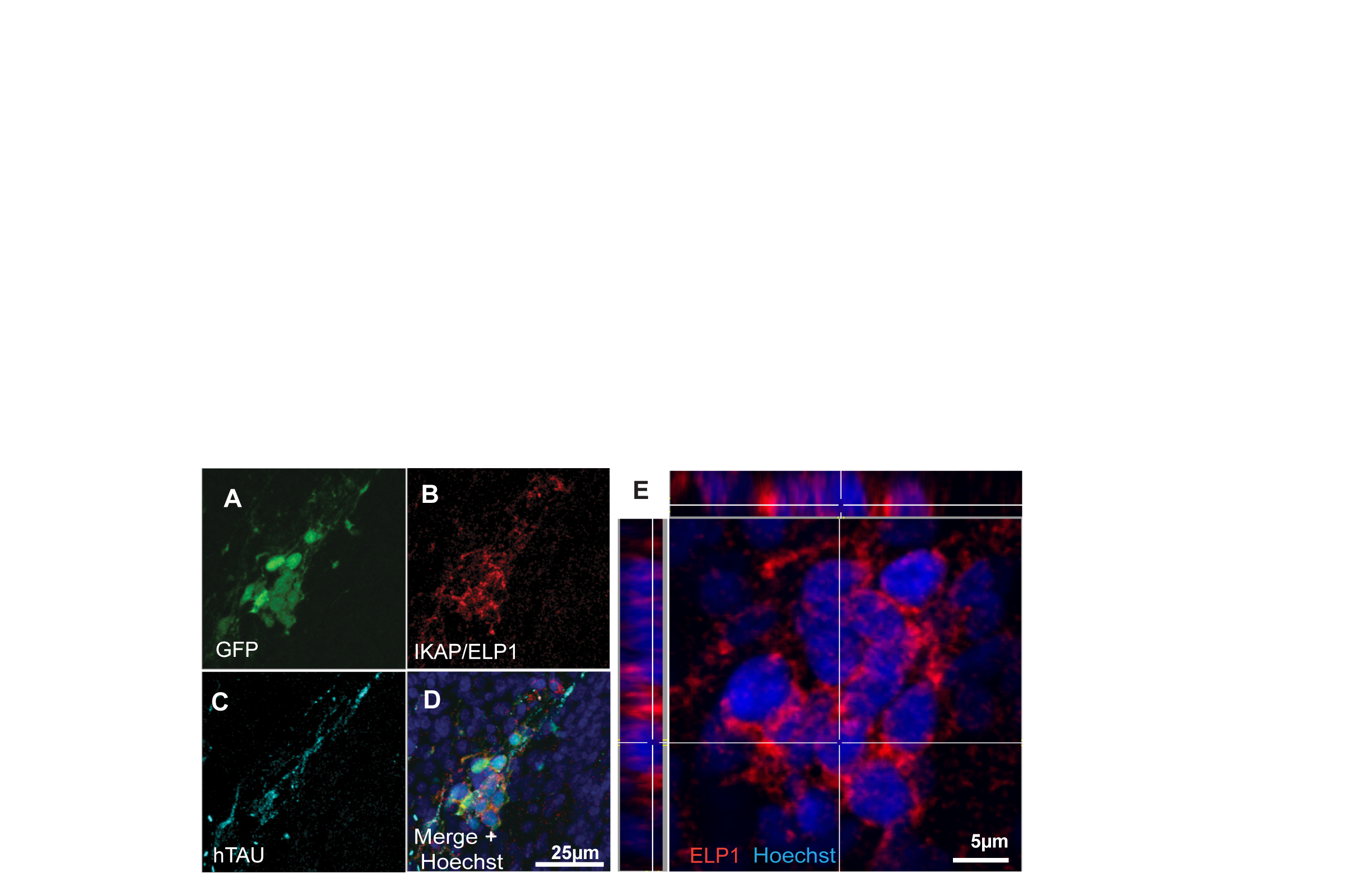

Supplement: Figure S3 — IKAP/hELP1 in hNPs derived PNS neurons in vivo. (A–E) Confocal micrographs showing the expression of IKAP/hELP1 in GFP+ together with the expression of human specific TAU (hTAU) in hNPs derived PNS neurons in the spinal cord of the chick embryo after 7 days of implantation. (E) Magnified area of the image in (A–D) showing orthogonal analysis of IKAP/hELP1 localization mainly in the cytosol of human PNS neurons in vivo. (2.41 MB TIF) [file pone.0009290.s003.tif]

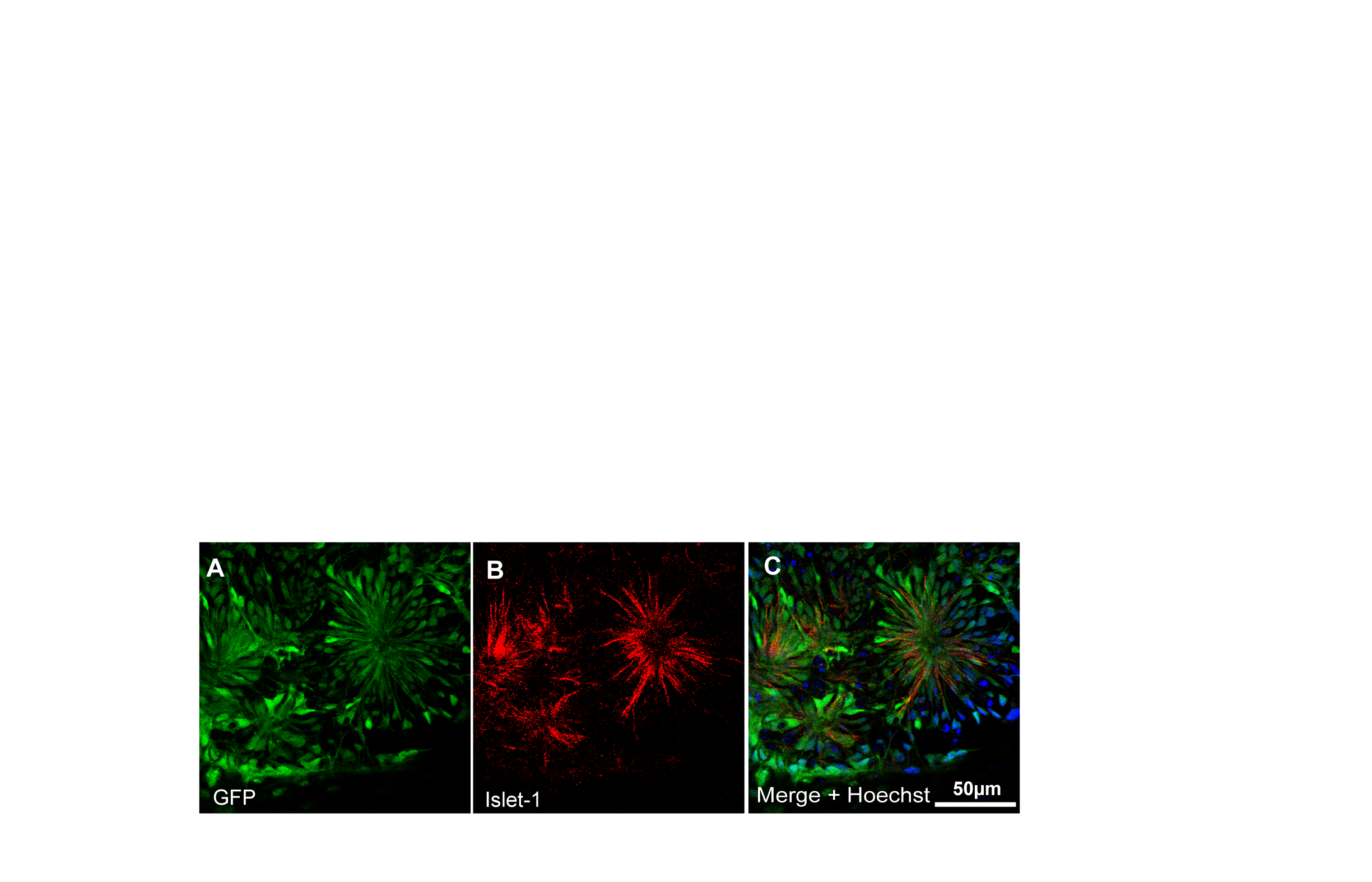

Supplement: Figure S4 — Five weeks hNPs differentiation culture shows rosettes formation with miss localization of Islet-1. (A–C) Five weeks old hNPs expressing GFP was plated on laminin coated surface for 12 days in culture showing typical rosette structure (A). Note that Islet-1 expression (B) in these cells is cytoplasmic and not nuclear as expected, which may indicate a more neuronal precursor state rather a mature neuron as shown in similar experiments with 8 weeks old hNPs (see Figure 2). (1.25 MB TIF) [file pone.0009290.s004.tif]
